# Circulating microRNA (miRNA) Expression Profiling in Plasma of Patients with Gestational Diabetes Mellitus Reveals Upregulation of miRNA miR-330-3p

**DOI:** 10.3389/fendo.2017.00345

**Published:** 2017-12-12

**Authors:** Guido Sebastiani, Elisa Guarino, Giuseppina Emanuela Grieco, Caterina Formichi, Chiara Delli Poggi, Elena Ceccarelli, Francesco Dotta

**Affiliations:** ^1^Diabetes Unit, Department of Medicine, Surgery and Neurosciences, University of Siena, Siena, Italy; ^2^Fondazione Umberto di Mario, Toscana Life Sciences, Siena, Italy; ^3^Azienda Ospedaliera Universitaria Senese, Siena, Italy

**Keywords:** microRNAs, plasma, gestational diabetes, biomarkers, miR-330-3p

## Abstract

Gestational diabetes mellitus (GDM) is characterized by insulin resistance accompanied by low/absent beta-cell compensatory adaptation to the increased insulin demand. Although the molecular mechanisms and factors acting on beta-cell compensatory response during pregnancy have been partially elucidated and reported, those inducing an impaired beta-cell compensation and function, thus evolving in GDM, have yet to be fully addressed. MicroRNAs (miRNAs) are a class of small endogenous non-coding RNAs, which negatively modulate gene expression through their sequence-specific binding to 3′UTR of mRNA target. They have been described as potent modulators of cell survival and proliferation and, furthermore, as orchestrating molecules of beta-cell compensatory response and function in diabetes. Moreover, it has been reported that miRNAs can be actively secreted by cells and found in many biological fluids (e.g., serum/plasma), thus representing both optimal candidate disease biomarkers and mediators of tissues crosstalk(s). Here, we analyzed the expression profiles of circulating miRNAs in plasma samples obtained from *n* = 21 GDM patients and from *n* = 10 non-diabetic control pregnant women (24–33 weeks of gestation) using TaqMan array microfluidics cards followed by RT-real-time PCR single assay validation. The results highlighted the upregulation of miR-330-3p in plasma of GDM vs non-diabetics. Furthermore, the analysis of miR-330-3p expression levels revealed a bimodally distributed GDM patients group characterized by high or low circulating miR-330 expression and identified as GDM-miR-330^high^ and GDM-miR-330^low^. Interestingly, GDM-miR-330^high^ subgroup retained lower levels of insulinemia, inversely correlated to miR-330-3p expression levels, and a significant higher rate of primary cesarean sections. Finally, miR-330-3p target genes analysis revealed major modulators of beta-cell proliferation and of insulin secretion, such as the experimentally validated genes E2F1 and CDC42 as well as AGT2R2, a gene involved in the differentiation of mature beta-cells. In conclusion, we demonstrated that plasma miR-330-3p could be of help in identifying GDM patients with potential worse gestational diabetes outcome; in GDM, miR-330-3p may directly be transferred from plasma to beta-cells thus modulating key target genes involved in proliferation, differentiation, and insulin secretion.

## Introduction

Gestational diabetes mellitus (GDM) is defined as any degree of carbohydrate intolerance, with onset or first recognition during second or third trimester of pregnancy (1). The prevalence in the world is increasing, and approximately 7% of all pregnancies are complicated by GDM (1), with major risk factors identified in previous history of GDM, history of macrosomia, familiarity for type 2 diabetes (T2D), elevated maternal age, and pre-pregnancy obesity (2, 3).

The pathophysiology of GDM is still not fully characterized. The inadequate β-cell adaptation to peripheral insulin resistance, characterizing second and third trimesters of gestation, is likely to be the main cause of GDM, even though the molecular mechanisms of such failure are mostly unknown (4). During normal pregnancy, glucose homeostasis is maintained by a compensatory increase in insulin secretion, associated with hypertrophy and/or hyperplasia of β-cells (5): it is supposed that several molecules, like placental hormones, play a central role in these adaptive changes (6), thus contributing to gene expression changes necessary to beta-cells in order to fulfill the compensatory request. Moreover, such compensatory phenomenon involves several specific beta-cell factors acting downstream these signaling molecules, such as the transcription factors MAFA and/or PDX1 whose expression strengthen and characterizes beta-cell phenotype maintenance (7, 8).

MicroRNAs (miRNAs) are endogenous ~19–24 nt small non-coding RNAs that play an important role in the modulation of gene expression (9). Indeed, through base pairing to partially complementary sites in the untranslated region of mRNAs, miRNAs can negatively modulate expression and translation of these molecules (10), thus being key factors in gene expression regulation. Each miRNA can potentially modulate multiple genes, whereas a single gene can be targeted by several miRNAs (11). Such complexity justifies the implication of miRNAs in virtually every cellular process, as well as in development or differentiation, regulation of cell cycle (12), and immune system homeostasis (13). Furthermore, miRNAs have been demonstrated to be involved in multiple sides of beta-cell function and differentiation (14), both in normal and diabetic conditions, as well as in beta-cell compensatory process during pregnancy.

Despite their function as regulators of gene expression, recent studies demonstrated that miRNAs are not exclusively intracellular, but also extracellular, being present in a cell-free circulating form in many different biological fluids, including serum or plasma (15). These miRNAs can be associated with proteins (vesicles-free) or held inside membranous vesicles such as shedding vesicles or exosomes. Importantly, extracellular circulating miRNAs have been found aberrantly expressed in the bloodstream during the course of many diseases (16), and some evidences suggested a potential role for miRNAs in cell–cell communication during pathological processes, particularly those packaged onto exosomes (17, 18). Furthermore, a putative use of circulating miRNAs as diagnostic, prognostic, and therapeutic biomarkers of many different diseases, have been strongly suggested, as they can be easily detected and measured from body fluids (19).

Recently, several studies have evaluated the expression of circulating miRNAs (plasma/serum) in diabetes, suggesting their putative use as early biomarkers of this group of chronic metabolic diseases (20). As a matter of fact, circulating miRNAs have been associated with β-cell mass and function and with immune system homeostasis, that certainly represent major players in diabetes pathogenesis (21–23). Regarding GDM, there is a paucity of data defining the expression and the diagnostic utility of circulating miRNAs in this important complication of pregnancy (24). Surely, the understanding of miRNAs function could improve knowledge about etiology and pathophysiology of GDM, while the characterization of circulating miRNAs expression could represent an important tool in order to diagnose GDM earlier than current methods (25). To the time of writing, only two groups have investigated the expression of circulating miRNAs in diabetic and non-diabetic pregnant women using high-throughput technologies. Zhao et al. have evaluated miRNAs profiling in serum collected at 16th–19th gestational weeks: three miRNAs (miR-132, miR-29a, and miR-222) were significantly decreased in GDM pregnant women with respect to the controls (CTR) (26). Similarly, Zhu et al. have evaluated miRNAs expression in plasma collected at 16th–19th gestational weeks: five miRNAs (hsa-miR-16-5p, hsa-miR-17-5p, hsa-miR-19a-3p, hsa-miR-19b-3p, and hsa-miR-20a-5p) were upregulated in diabetic pregnant women with respect to CTR (27). Although informative, the results of these two studies are discordant, probably due to the different samples analyzed (serum vs plasma) and to the different procedures used.

Due to the paucity of data on circulating miRNAs in GDM and to the presence of discordant data, we aimed at evaluating circulating miRNAs expression profiles in plasma of diabetic vs non-diabetic pregnant women during the third trimester of gestation, with the final goal to identify new potential diagnostic/prognostic GDM biomarkers as well as to potentially elucidate part of the complex mechanisms involved in the onset of GDM.

## Materials and Methods

### Patients and Blood Samples

The patients were recruited in the Diabetes out-patient Unit in Siena University Hospital. All the patients performed a 75-g oral glucose tolerance test at 16–19 weeks or 24–28 weeks of pregnancy, and GDM was diagnosed if one of the three glucose concentrations measured was above the cutoff values (fasting plasma glucose ≥5.1 mmol/l; 1-h plasma glucose ≥10 mmol/l; 2-h plasma glucose ≥8.5 mmol/l), according to Italian guidelines. Peripheral blood samples were collected from 21 women with GDM and 10 pregnant non-diabetic CTR at 24–33 weeks of pregnancy to evaluate miRNAs expression and laboratory parameters (glycemia, cholesterol, triglycerides, insulin levels, HbA_1c_, creatinine, urinary albumin, osteocalcin, 25-OH vitamin D, calcitonin, and serum calcium). Moreover, during the third trimester of pregnancy, all the 31 participants performed an obstetric echography to evaluate fetal parameters (biparietal diameter, cranial circumference, abdominal circumference, femur length, and fetal weight). Patients’ main clinical parameters are reported and summarized in Table 1. This study was carried out in accordance with the required local ethical recommendations. All procedures followed were in accordance with the ethical standards of the responsible committee on human experimentation (institutional and national) and with the Helsinki Declaration of 1975, as revised in 2013. Informed consent was obtained from all patients for being included in the study.

**Table 1:** Clinical characteristics (maternal and fetal) of the study population.

Clinical parameter	microRNAs profiling cohort		Validation cohort	
	Non-diabetic pregnant patients (*n* = 4)	GDM patients (*n* = 4)	Non-diabetic pregnant patients (*n* = 10)	GDM patients (*n* = 21)
**Maternal characteristics**				
Age (years)	35.25 ± 3.86	35.00 ± 3.91	32.80 ± 5.16	35.57 ± 5.63
BMI (kg/m^2^)	20.40 ± 0.77	21.75 ± 1.11	22.85 ± 3.46	25.25 ± 4.00
Glycemia (mg/dl)	67.00 ± 0.00	80.00 ± 2.16*	69.33 ± 8.35	80.32 ± 7.64**
Insulinaemia (mUI/ml)	4.30 ± 0.69	10.85 ± 4.05*	7.36 ± 3.29	11.84 ± 6.03**
HbA1c (%)	4.76 ± 0.32	5.175 ± 0.15	4.94 ± 0.26	5.19 ± 0.32
Total cholesterol (mg/dl)	267.00 ± 58.50	276.75 ± 79.81	259.75 ± 39.47	271.84 ± 53.65
Triglycerides (mg/dl)	187.33 ± 63.32	160.75 ± 18.12	173.87 ± 59.75	218.21 ± 57.42
Urinary Albumin (mg/l)	4.40 ± 2.94	2.67 ± 0.65	7.378 ± 10.334	16.60 ± 54.97
Creatinine (mg/dl)	0.52 ± 0.11	0.55 ± 0.08	0.56 ± 0.07	0.53 ± 0.07
Osteocalcin (ng/ml)	21.27 ± 2.82	15.07 ± 4.26	18.86 ± 3.93	14.53 ± 3.50
25-(OH)D3 (ng/ml)	16.20 ± 3.16	20.77 ± 6.70	17.76 ± 8.38	17.54 ± 7.82
Calcitonin (pg/ml)	1.57 ± 1.52	3.67 ± 4.99	1.73 ± 1.92	3.02 ± 3.07
Ca^++^ (mg/dl)	8.74 ± 0.18	9.35 ± 0.46	8.72 ± 0.21	9.01 ± 0.37

**Fetal characteristics**				
FW (g)	2,274.00 ± 311.19	2,167.08 ± 302.23	2,126.50 ± 406.12	2,050.37 ± 238.29
FBD (mm)	86.40 ± 2.71	79.20 ± 2.45	84.16 ± 3.98	82.34 ± 2.97
FHC (mm)	310.52 ± 16.66	304.22 ± 15.75	304.87 ± 16.08	300.82 ± 8.49
FAC (mm)	296.97 ± 11.00	285.65 ± 19.13	288.27 ± 17.26	284.63 ± 13.42
FFL (mm)	65.25 ± 2.18	61.77 ± 5.64	63.57 ± 3.77	63.53 ± 3.36

*FW, fetal weight; FBD, fetal biparietal diameter; FHC, fetal head circumference; FAC, fetal abdominal circumference; FFL, fetal femur length; GDM, gestational diabetes mellitus. Data are reported as mean ± SD*.

*p < 0.05 GDM vs non-diabetic pregnant patients (profiling cohort).

**p < 0.05 GDM vs non-diabetic pregnant patients (validation cohort).

### Blood Samples Processing

The blood samples were processed according to a standardized operating procedure to collect plasma for miRNAs analysis. The blood was drawn in BD Vacutainer K_2_-EDTA tubes and processed within 2 h from collection.

The whole blood was separated into plasma and cellular fractions by centrifugation at 1,800*g* for 10 min at room temperature; plasma was collected in RNAse-free tubes and further centrifuged at 1,200*g* for 20 min at 10°C to completely remove contaminant cells. Finally, plasma samples were aliquoted to avoid freeze–thaw cycles and finally stored at −80°C up to the RNA extraction.

### RNA Extraction

Plasma samples were thawed on ice and then further centrifuged 3,000*g* for 5 min at 4°C in order to completely remove cell debris. MiRNeasy miRNA extraction kit (Qiagen, Hilden, Germany) was used to extract RNA from 50 µl of plasma from each patient, by adding 1,200 µl of Trizol LS (Lifetechnologies, CA, USA) and finally eluted in 30 µl of nuclease-free water. Moreover, 25 pmol of the spike-in control ath-miR-159a were added to each sample, in order to control variations in RNA extraction procedures.

### TaqMan miRNA Array Profiling Analysis

TaqMan miRNA Human Array Panel A platform (Lifetechnologies, CA, USA) was adopted to profile the expression the expression of 384 miRNAs in as low as 50 µl of plasma, following manufacturer’s instructions. RNA was reverse-transcribed using “Megaplex RT primers Pool-A” (Lifetechnologies, CA, USA); briefly, 3 µl of extracted RNA from 50 µl of each plasma sample were added to 0.80 µl of 10× Megaplex RT Primers, 0.20 µl of 100 mM dNTPs, 1.50 µl of 50 U/μl Multiscribe RT, 0.80 µl of 10× RT Buffer, 0.90 µl of 25 mM MgCl_2_, 0.10 µl of 20 U/μl RNAse Inhibitor, and 0.20 µl of H_2_O. The product of this reaction was incubated for 40 cycles at 16°C for 2 min, 42°C for 1 min, and 50°C for 1 s, and then at 85°C for 5 min. Afterward, the synthesized cDNA was preamplified using “Megaplex Preamp primers Pool-A”: 2.5 µl of cDNA from each sample were added to 12.5 µl of 2× TaqMan Preamp Master Mix, 2.5 µl of 10× Preamp Primers A V.2.1, and 7.5 µl of H_2_O. The product of this reaction was incubated at 95°C for 10 min, at 55°C for 2 min, and at 72°C for 2 min, then for 12 cycles at 95°C for 15 s and 60°C for 4 min and, finally, at 99°C for 10 min. Finally, preamplified cDNA was diluted 1:4 in 0.1× Tris-EDTA pH8.0 to obtain a final volume of 100 µl.

The reaction mix for each microfluidic card was prepared adding 360 µl of H_2_O and 450 µl of TaqMan Universal PCR Master Mix 2× to 90 µl of diluted and preamplified cDNA. The product of this reaction was incubated at 95°C for 10 min, followed by 40 cycles of 95°C for 15 s and 60°C for 1 min. The real-time PCR instrument ViiA7 (Lifetechnologies, CA, USA) was used to perform the reactions.

Resulting data were analyzed and exported using Expression Suite 1.2.1 software (Lifetechnologies, CA, USA). Analysis was performed by using 2^−ΔΔCt^ method following normalization with both Global Mean Normalization method and NormFinder search for most stable endogenous miRNAs (miR-374, miR-320).

Hierarchical clustering analysis plot was computed in order to obtain a global view of miRNAs expression levels among eight plasma samples analyzed and to identify clustered group of miRNAs. Differentially expressed miRNAs were identified by performing a volcano plot analysis by applying a cutoff fold change of 2.0 and a statistical cutoff of *p* < 0.05 using Student’s t test and Mann–Whitney statistical non-parametrical *U* test. Hierarchical Clustering analysis plot and Volcano Plot were elaborated using Spotfire 5.0 (Tibco) and GraphPad 6.0, respectively.

### Single Assay qRT Real-time PCR

The expression of miRNAs miR-330-3p and miR-548c was analyzed in all the 31 plasma samples through single assay qRT real-time PCR using TaqMan miRNA assay primers (Lifetechnologies, CA, USA). RNA was reverse-transcribed using “Megaplex RT primers Pool-A” protocol and preamplified using “Megaplex Preamp primers Pool-A” (see above). In each well, 5 µl of preamplified cDNA (diluted 1:40) were added to 15 µl of reaction mix composed of 10 µl TaqMan Universal Master Mix, 1 µl of TaqMan miRNA expression assay, 4 µl of H_2_O. The reaction was incubated at 95°C for 10 min, followed by 40 cycles of 95°C for 15 s and 60°C for 1 min. Data analysis was performed by using 2^−ΔCt^ method; samples with resulting raw cycle-threshold (Ct) >35.0 were considered as not detected/expressed. miRNAs miR-320 and miR-374a were adopted to normalize the values of detected miRNAs of interest.

### Statistical Analysis

Differences in expression levels of miRNAs, in clinical characteristics (age, BMI), in laboratory parameters and in obstetric parameters were evaluated by Student’s t test, non-parametric Mann–Whitney *U* test or Fisher’s Exact test. The correlation between expression levels of hsa-miR-330-3p and insulin levels was evaluated by Spearman non-parametric test. A *p*-value < 0.05 was considered as statistically significant. GraphPad 5.1 was adopted for statistical analysis of the data.

### Target Genes Identification and Bioinformatic Analysis

In order to retrieve predicted or previously experimentally validated miRNAs target genes, we used Targetscan Human 7.0^1^ (28) and miRTarbase^2^ (29), respectively.

Interaction network analysis was performed by using BioGRID 3.4 tool^3^ (30) in order to verify all the potential interactions of identified target genes and potentially function mediators.

## Results

### Plasma miRNAs Expression Profiles in GDM Patients

In an attempt to verify the potential altered expression of circulating miRNAs in plasma of GDM patients, we performed an unbiased TaqMan Array profiling by analyzing 384 miRNAs in a discovery cohort composed of plasma samples derived from *n* = 4 non-diabetic pregnant women (24–33 weeks of gestation; age 35.25 ± 3.86 years; BMI 20.40 ± 0.77 kg/m^2^) and *n* = 4 GDM patients (24–28 weeks of gestation; age 35.00 ± 3.9 years; BMI 21.5 ± 1.11 kg/m^2^) (Table 1—profiling cohort). A total of 202 miRNAs were detected (raw Ct cutoff < 35.0) in at least one sample/group, with a global mean miRNAs detection rate between 40 and 50% (Figure S1A in Supplementary Material). Since global mean normalization strategy for miRNAs expression data analysis has been indicated as the most reliable method to detect changes in circulating miRNA expression levels, we adopted such strategy to analyze resulting raw data; indeed such approach showed a good degree of stability across samples. However, in order to detect single miRNAs which can be reliably used as plasma miRNA normalization factors for subsequent single assay validation stage, we looked for two most stable plasma miRNAs among 202 detected across the eight samples analyzed; using the algorithm NormFinder, we identified miR-320 and miR-374a as the most stably expressed miRNAs (Figure S1C in Supplementary Material) and with comparable stability respect to the global mean normalization.

**Figure 1:**
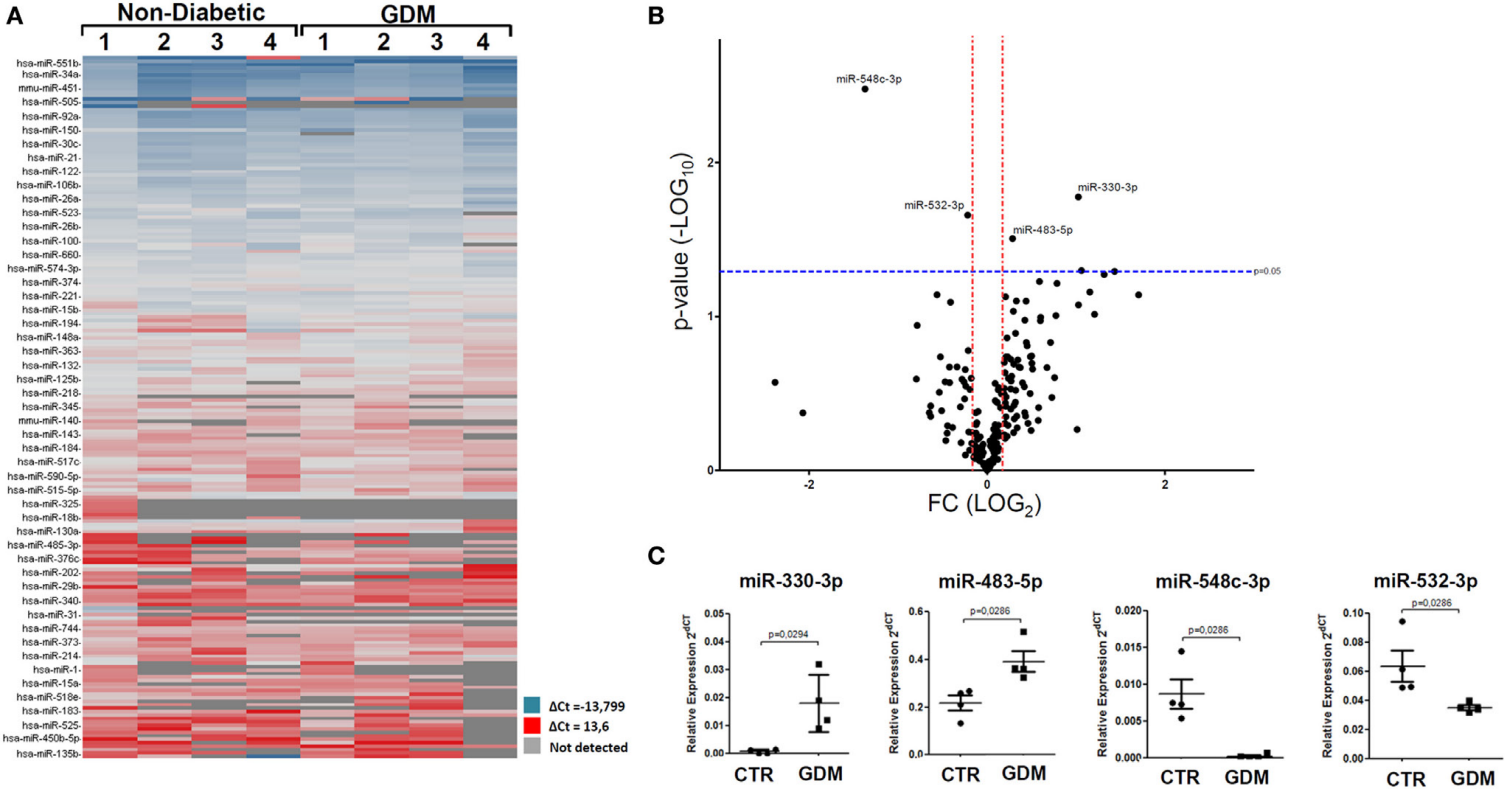
Taqman microRNAs (miRNAs) Array profiling in plasma of gestational diabetes mellitus (GDM) patients. **(A)** Hierarchical Clustering Heatmap analysis of miRNAs detected in at least one sample/group. *n* = 4 non-diabetic controls (CTR) and *n* = 4 GDM patients are reported. miRNAs expression levels are reported as scale colors based on dCT expression [blue (high expression): dCT = −13.7; red (low expression): dCT = 13.6; grey (not detected)]. **(B)** Volcano plot analysis showing differentially expressed miRNAs; single black dot represents miRNAs positioned based on the relative fold change and *p*-values. Fold change cutoff (red lines) was set at 2.0-fold while *p*-values cutoff (blue line) was set at 0.05 based on Student’s *t*-test on normally distributed dCT. **(C)** Results reported as dot plot graph showing miRNAs differentially expressed; extrapolated values from Taqman array profiling are reported as normalized 2^−dCT^ together with mean ± SEM and *p*-values further elaborated using non-parametric Mann–Whitney *U* test (*p* < 0.05).

Overall, resulting data are reported in the hierarchical clustering heatmap analysis showing the expression levels of detected miRNAs analyzed using global mean normalization (Figure 1A). Since our aim was to identify potential miRNAs expression alterations in plasma of GDM patients respect to non-diabetic CTR, adopting a specific fold change (2.0-fold change upregulated/downregulated vs non-diabetics) and *p*-value cutoffs (*p* < 0.05 Student’s *t*-test on normally distributed ΔCt), we detected the differential expression of four miRNAs, as demonstrated by Volcano Plot analysis (Figure 1B). Namely, we identified miR-330-3p and miR-483-5p (upregulated in GDM vs non-diabetic subjects) and miR-548c-3p and miR-532-3p (downregulated in GDM vs non-diabetic subjects) (Figures 1B,C). As shown in Volcano plot, miR-330-3p and miR-548c-3p resulted the most differentially expressed miRNAs in GDM vs non-diabetic pregnant women (miR-330-3p: 11.1-fold increase; miR-548c-3p: 25.3-fold decrease; miR-483-5p: 2.01-fold increase; miR-532-3p: 2.1-fold decrease) and then taken into consideration for further analyses involving a second patients cohort.

**Figure 2:**
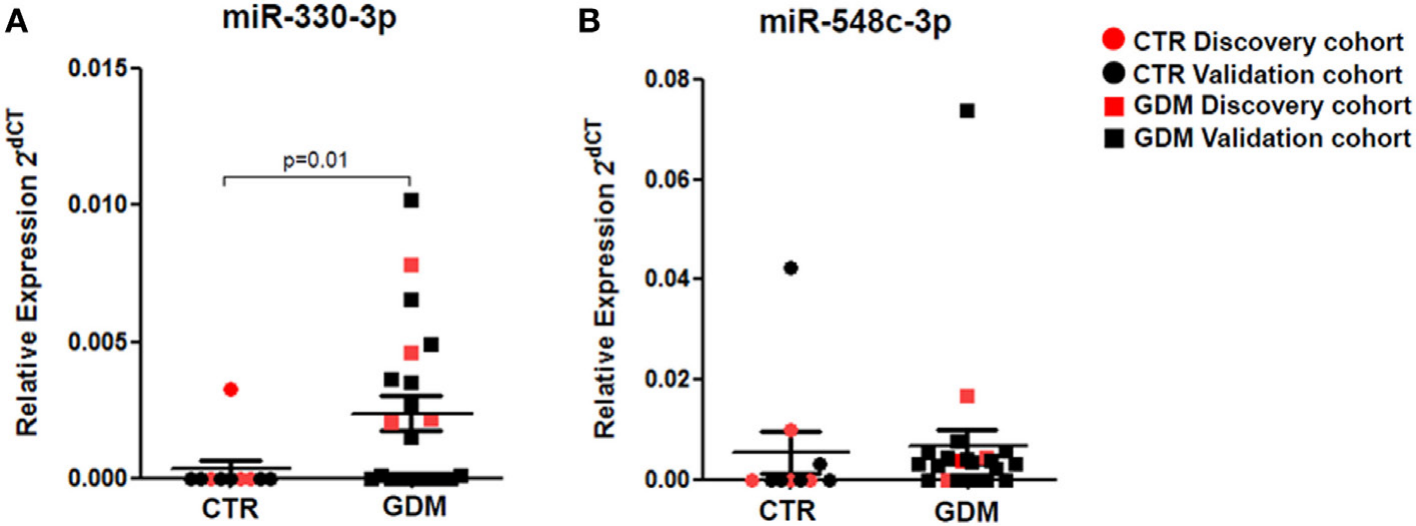
RT-PCR Single assay validation of miR-330-3p and miR-548c-3p. Single assay RT-real-time PCR validation of **(A)** miR-330-3p and **(B)** miR-548c-3p on *n* = 10 non-diabetic controls (CTR) and *n* = 21 gestational diabetes mellitus (GDM) patients. Red dots represent the discovery cohort analyzed in the miRNA profiling stage while the black dots represent the validation cohort. Data are reported as normalized 2^−dCT^ values together with mean ± SEM. Mann–Whitney *U* test, *p* < 0.05.

Stem-loop RT-real-time PCR was adopted to perform single assay validation analysis of miRNAs miR-330-3p and miR-548c-3p. Such validation analysis was performed by including a second cohort of patients, composed of additional samples from non-diabetic and GDM patients (Table 1—validation cohort). Therefore, the expression of such miRNAs were finally evaluated in a total of *n* = 10 non-diabetic pregnant women and *n* = 21 GDM patients. The results highlighted a significant increased expression of miR-330-3p (*p* = 0.01, Mann–Whitney *U* test) in plasma of GDM vs non-diabetic subjects (Figure 2A) while no significant differences were observed in miR-548c-3p expression levels (Figure 2B).

### Plasma Levels of miR-330-3p Define Two GDM Patient Subgroups

**Figure 3:**
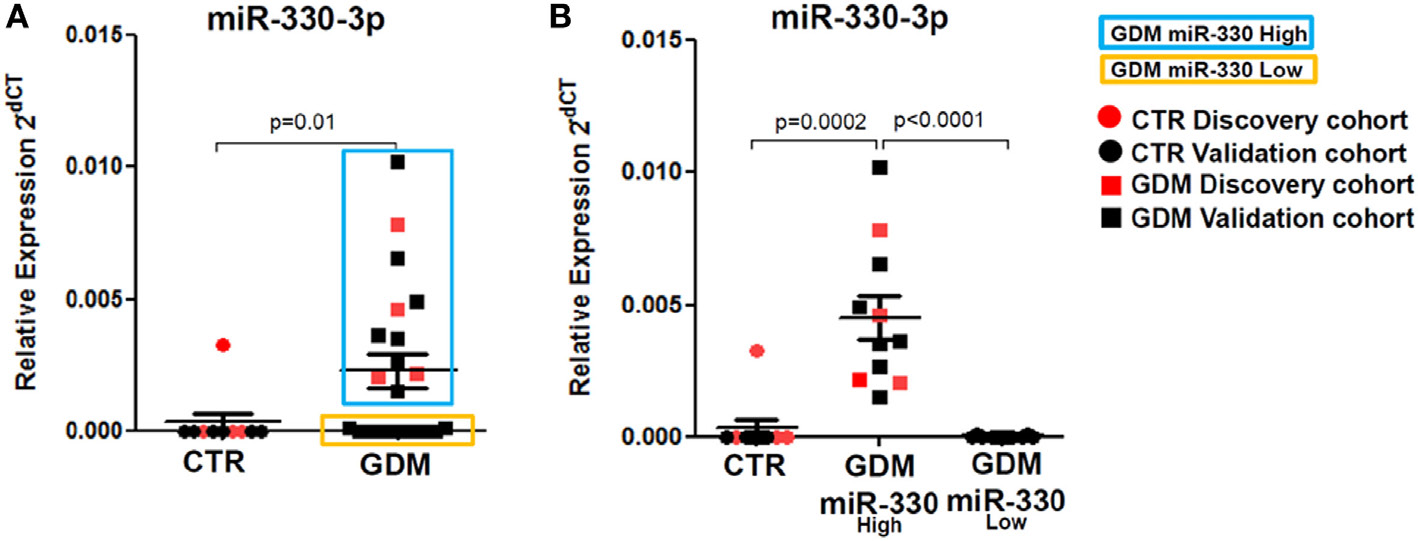
Plasma miR-330-3p identifies two subpopulations of gestational diabetes mellitus (GDM) patients. **(A)** Dot plot graphs depicting the expression of miR-330-3p, which identifies two different GDM subpopulations: miR-330^high^ (blue rectangle), miR-330^low^ (yellow rectangle). **(B)** Expression of plasma miR-330-3p in non-diabetic controls (CTR) (*n* = 10), GDM-miR-330^high^ (*n* = 11), and GDM-miR-330^low^ (*n* = 10). Data are reported as normalized 2^−dCT^ values together with mean ± SEM. Mann–Whitney *U* test, *p* < 0.05.

Interestingly, by analyzing miR-330-3p expression data, we detected a clear-cut separation within GDM patients group, characterized by *n* = 11 patients with high miR-330-3p expression (GDM miR-330^high^) and *n* = 10 GDM patients with low/null miR-330-3p expression (GDM miR-330^low^) (Figure 3A); miR-330-3p levels significantly differed between the two subgroups and respect to non-diabetic subjects as well (Figure 3B). Based on miR-330-3p expression, GDM patients group resulted bimodally distributed, thus clearly distinguishing two GDM subgroups.

**Figure 4:**
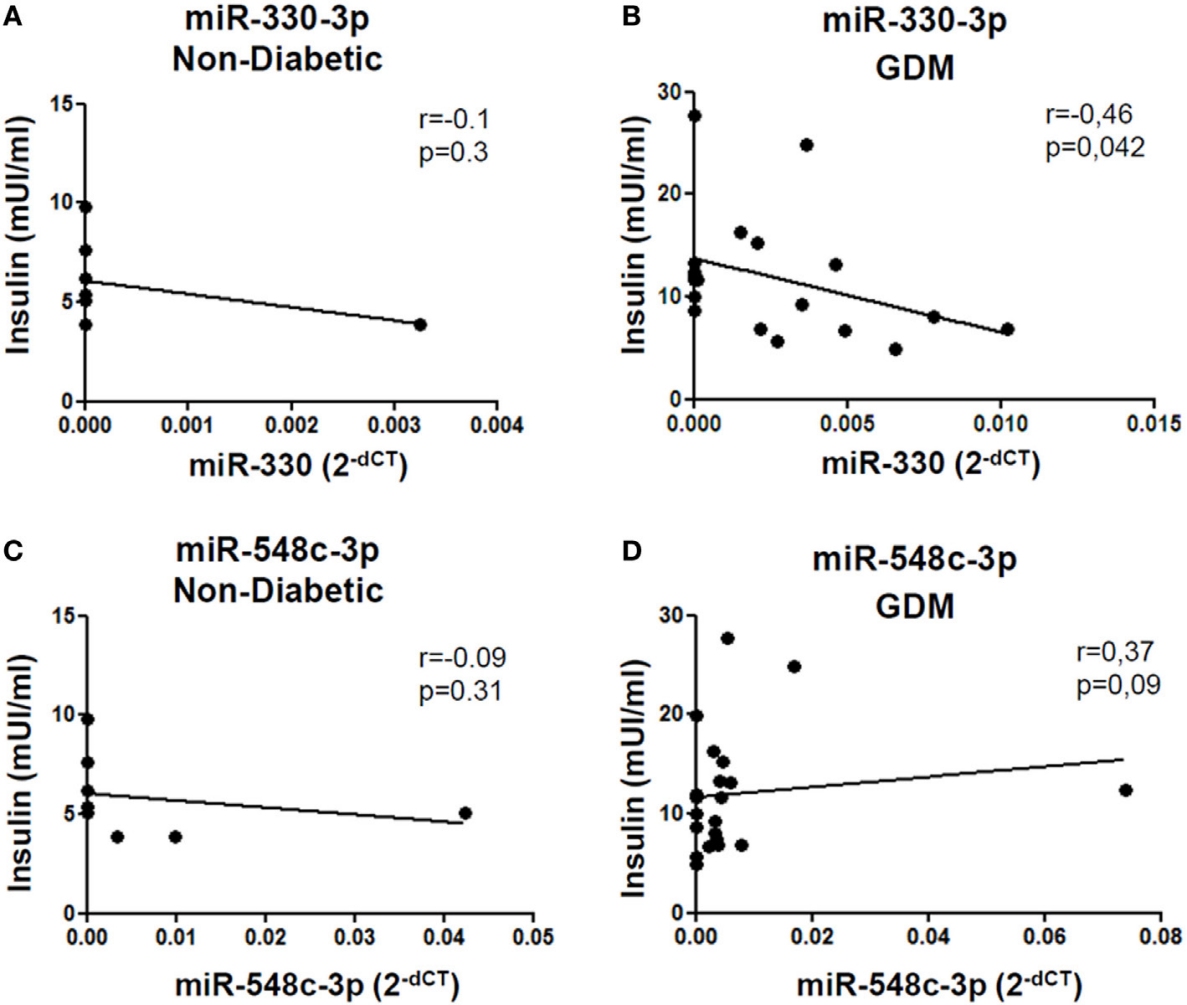
miR-330-3p expression correlates with insulinemia in gestational diabetes mellitus (GDM) patients. Correlation analysis between miR-330-3p and miR-548c-3p expression levels, reported as normalized 2^−dCT^ values, and insulinemia, reported as mUI/ml, in **(A,C)** non-diabetic controls (*n* = 8) and in **(B,D)** GDM patients (*n* = 21). Spearman *R* test was performed to evaluate *r*-values and *p*-values (*p* < 0.05).

Such peculiar bimodal distribution within GDM patients led us to verify whether potential differences in clinical parameters, maternal diabetic outcomes, and/or fetal outcomes may occur between miR-330^high^ and miR-330^low^ GDM patients. First, no differences were detected between the two subgroups in terms of age and BMI (Table 2). Interestingly, we observed that insulinemia (measured at onset) was lower in miR-330^high^ respect to miR-330^low^ GDM patients (Table 2), while, as expected, both GDM subgroups retained higher levels of insulinemia respect to non-diabetic subjects. More importantly, we observed that the expression of circulating miR-330-3p in GDM patients, but not in non-diabetics, was significantly inversely correlated to insulinaemia (Figure 4A), as an increased expression of circulating miR-330-3p resulted in lower insulinemia (*p* = 0.042) (Figure 4B). Correlation analysis of miR-548c-3p expression levels and insulinemia both in GDM and in non-diabetic subjects did not retrieve any significant results (Figures 4C,D).

**Table 2:** Clinical characteristics (maternal and fetal) of the study population subgrouped based on plasma miR-330-3p expression.

Clinical parameters	Non-diabetic pregnant patients (*n* = 10)	miR-330^high^ GDM patients (*n* = 11)	miR-330^low^ GDM patients (*n* = 10)
**Maternal characteristics**			
Age (years)	32.80 ± 5.16	36.55 ± 5.48	34.50 ± 5.87
BMI (kg/m^2^)	22.85 ± 3.46	24.90 ± 4.55	25.64 ± 3.49
Glycemia (mg/dl)	69.33 ± 8.35	81.18 ± 6.89	79.13 ± 8.90
Insulinaemia (mUI/ml)	7.36 ± 3.29	10.71 ± 6.09	13.40 ± 5.96
HbA1c (%)	4.94 ± 0.26	5.24 ± 0.35	5.13 ± 0.27
Total cholesterol (mg/dl)	259.75 ± 39.47	266.55 ± 58.89	279.13 ± 48.39
Triglycerides (mg/dl)	173.87 ± 59.75	207.91 ± 66.11	232.37 ± 42.89
Urinary albumin (mg/l)	7.378 ± 10.334	3.21 ± 1.89	35.01 ± 84.25
Creatinine (mg/dl)	0.56 ± 0.07	0.54 ± 0.06	0.52 ± 0.08
Osteocalcin (ng/ml)	18.86 ± 3.93	13.97 ± 3.89	14.96 ± 3.34
25-(OH)D3 (ng/ml)	17.76 ± 8.38	17.63 ± 7.24	17.44 ± 8.82
Calcitonin (pg/ml)	1.73 ± 1.92	2.67 ± 3.28	3.37 ± 2.99
Ca^++^ (mg/dl)	8.72 ± 0.21	9.14 ± 0.37	8.87 ± 0.34
Week of gestation at delivery (weeks)	39.80 ± 1.48	38.70 ± 1.41	39.50 ± 1.37

**Fetal characteristics**			
FW (g)	2,126.50 ± 406.12	2,096.36 ± 279.25	1,987.13 ± 163.46
FBD (mm)	84.16 ± 3.98	82.79 ± 3.85	81.71 ± 0.84
FHC (mm)	304.87 ± 16.08	301.20 ± 10.15	300.29 ± 6.13
FAC (mm)	288.27 ± 17.26	287.94 ± 15.42	280.09 ± 9.09
FFL (mm)	63.57 ± 3.77	63.64 ± 3.84	63.39 ± 2.80

*FW, fetal weight; FBD, fetal biparietal diameter; FHC: fetal head circumference; FAC, fetal abdominal circumference; FFL, fetal femur length; GDM, gestational diabetes mellitus. Data are reported as mean ± SD*.

Additionally, although not statistically significant, patients within GDM-miR-330^high^ subgroup retained an higher rate of predisposing T2D genetic background, having at least one first-degree relative affected by T2D and, furthermore, were more likely to require future insulin therapy respect to patients with low miR-330-3p expression (Table 3). Moreover, by measuring pregnancy outcomes, we observed a significant increased rate of cesarean sections in pregnant women within GDM-miR-330^high^ subgroup respect to GDM-miR-330^low^, due to advanced maternal age, previous cesarean sections and complications of pregnancy (e.g., maternal hydronephrosis, polyhydramnios, fetal macrosomia). This suggests a worse pregnancy outcome possibly due to a more aggressive diabetic phenotype characterized by increased circulating expression of miR-330-3p.

**Table 3:** Comparison between gestational diabetes mellitus (GDM) subgroups based on selected diabetic and pregnancy/pre-pregnancy outcomes.

	GDM miR-330^high^ (%)	GDM miR-330^low^ (%)	*p*-Value
Insulin therapy	5/11 (55)	2/10 (20)	0.36
T2D first-degree relatives	6/11 (66)	2/10 (20)	0.18
Cesarean sections	8/11 (88)	1/10 (10)	*0.0075*
Non spontaneous conception	4/11 (44)	0/10 (0)	0.092

*The following parameter are reported: requirement of insulin therapy during third trimester of gestation; differences in familiarity for type 2 diabetes (T2D) (evaluated in first-degree relatives); delivery through Cesarean section; conception through medically assisted procreation techniques (e.g., ICSI, IVF). The results are expressed both in number of the total and in percentage*.

*Italic font highlights significant p-values using Fisher’s exact test*.

### miR-330-3p Targets Several Genes Involved in Beta-Cell Compensatory Process and in Glucose Homeostasis

**Figure 5:**
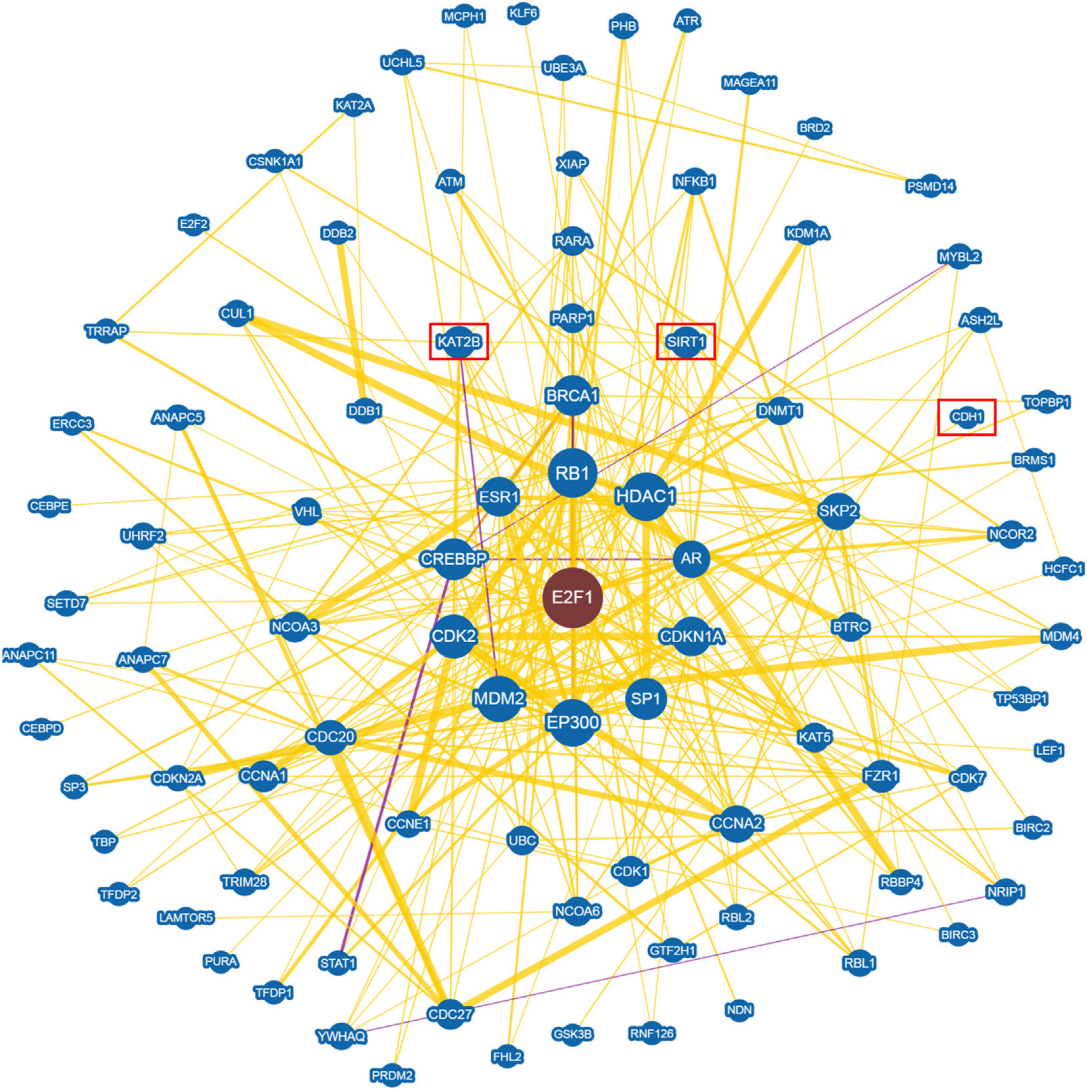
E2F1 Interaction networks analysis graph performed using Biogrid software tool. Interaction networks analysis between E2F1 and its reported protein interactors, show several factors involved in the control of proliferation. Yellow connection lines represent a physical interaction evidence while purple connection lines reports both a genetic and physical interaction. Only those interactions with three or more reported and published evidences are shown. Greater node size represents increased connectivity while thicker edges represent increased evidences. Red rectangles highlight interactors with a previously described role in beta-cell function.

**Figure 6:**
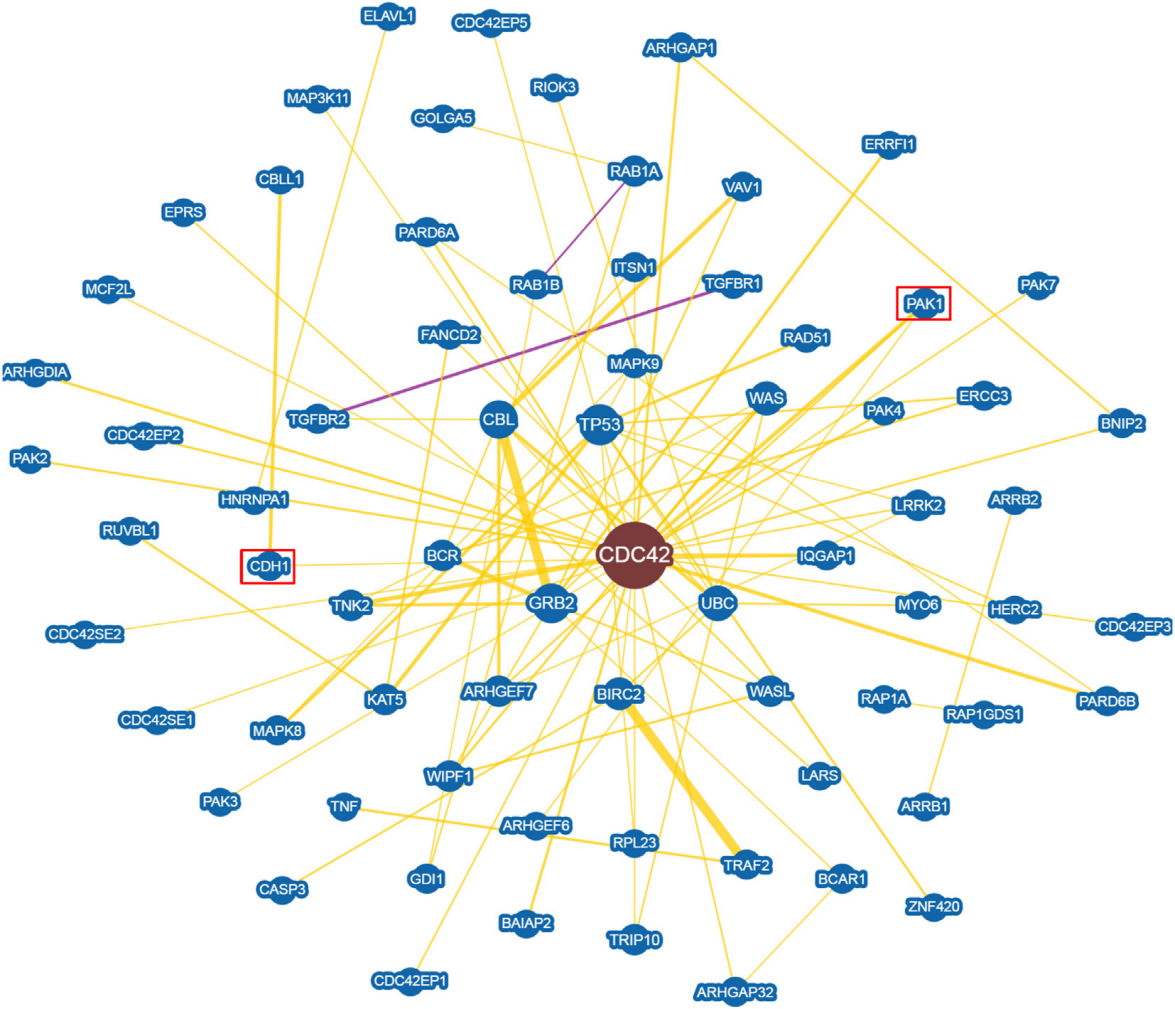
CDC42 Interaction networks analysis graph performed using Biogrid software tool. Interaction networks analysis between CDC42 and its reported protein interactors, show several factors involved in the control of proliferation. Yellow connection lines represent a physical interaction evidence while purple connection lines reports both a genetic and physical interaction. Only those interactions with three or more reported and published evidences are shown. Greater node size represents increased connectivity while thicker edges represent increased evidences. Red rectangles highlight interactors with a previously described role in beta-cell function.

An in depth analysis of the potential functions of miR-330-3p through the evaluation of its previously experimentally validated target genes, revealed that miR-330-3p targets two components of beta-cell function and proliferation machinery: E2F1 and CDC42 (Table 4) (31, 32). Both identified genes are involved in beta-cell growth and proliferation being activators of a series of proteins involved in cell cycle activation and cellular growth; interaction network analysis (Figures 5 and 6) shows all the previously dema consistent and significant number of interactions are toward factors highly involved in proliferation. However, the interaction network analysis revealed that several other interacting genes have been demonstrated to control beta-cell phenotype maintenance, such as KAT2B, CDH1, SIRT1, and PAK1 (Figures 5 and 6, highlighted in red). Furthermore, beside their role in cellular growth and proliferation, both E2F1 and CDC42 have been also demonstrated to be involved in glucose-stimulated insulin secretion, being prevalently involved in first- and second-phase insulin release, respectively (33–36).

**Table 4:** MicroRNA miR-330-3p validated and predicted target genes.

Official symbol	Official full name	Sequence accession ID	Predicted/validated	Function
E2F1	E2F transcription factor 1	NM_005225.2	Validated	Overexpression of E2F1 can stimulate beta-cell proliferation activity. E2F1^−/−^ mice have a reduced pancreatic size and are glucose intolerant due to impaired beta-cell proliferation
				Involved in insulin secretion (36)

CDC42	Cell division cycle 42	NM_001039802.1	Validated	Regulates signaling pathways that control several cellular functions including cell morphology, migration, endocytosis, and cell cycle progression. Depletion of Cdc42 from mouse isolated islets results in the selective loss of second-phase insulin release (35)

AGTR2	Angiotensin II receptor type 2	NM_000686.4	Predicted	G-protein coupled receptor 1 family that functions as a receptor for angiotensin II. AGTR2 receptor is involved in beta-cell differentiation (37, 38)

TFEB	Transcription Factor EB	NM_001167827.2	Predicted	Plays a central role in the signal transduction processes required for normal vascularization of placenta (39)

EIF4EBP2	Eukariotic Translation Initiation Factor 4E binding protein	NM_004096.4	Predicted	Regulation of protein production through these gene products have been implicated in beta-cell proliferation (40, 41)

IRS-4	Insulin receptor substrate 4	NM_003604.2	Predicted	Involved in cell growth and glucose homeostasis (42, 43)

Finally, among predicted target genes, we identified other key factors, which could be involved in beta-cell proliferation and differentiation, thus potentially being negatively modulated by increased expression of miR-330-3p (Table 4).

## Discussion

Circulating miRNAs have been suggested as potential biomarkers of several diseases being potentially secreted in biological fluids by virtually all cell types (44). Furthermore, they have been observed as mediators of tissue crosstalk, acting both in physiological conditions as well as in diseases, thus being suggested as new “hormones” (45). Consequently, investigating circulating miRNAs expression may potentially reveal both new biomarkers of disease as well as suggest new pathogenic mechanisms.

Regarding GDM, two previous studies analyzed circulating miRNAs expression profiles in plasma and serum, adopting high-throughput techniques. However, the results between the two studies were discordant, possibly due to different types of biological samples analyzed (serum vs plasma) as well as potential differences in pre-analytical sample processing (46). Indeed, miRNAs expression in serum or plasma is strongly influenced by small changes in pre-analytical steps and several strict guidelines need to be followed in order to reduce variability across samples and to finally identify reliable biomarkers (47). In the present study, we analyzed the expression profile of 384 miRNAs in plasma samples derived from non-diabetic pregnant women and GDM pregnant patients with the aim: (i) to detect new biomarkers of gestational diabetes; (ii) to suggest new pathogenic mechanisms leading to gestational diabetes onset. Importantly, samples were collected using a standardized protocol specifically following all the necessary procedures to reliably identify potentially altered miRNA expression. A two-step analysis was adopted in order to identify differentially expressed miRNAs: (1) a high-throughput approach involving a selected subpopulation of GDM patients and CTR (age and BMI matched), followed by (2) an extended validation stage in an additional cohort aimed at characterizing the expression of single miRNAs previously detected in the first profiling step. Adopting such approach we finally confirmed the differential expression of miR-330-3p, which resulted significantly upregulated in GDM plasma samples vs non-diabetic CTR. MiR-330-3p expression levels were inversely correlated to insulinemia and such correlation was restricted to GDM patient. These results may suggest a new potential connection between miR-330-3p expression and diabetic state. More interestingly, we identified two subpopulations of GDM patients characterized by different levels of miR-330-3p: GDM-miR-330^high^ and GDM-miR-330^low^. The GDM patients group retaining higher plasmatic levels of miR-330-3p showed signs of a more aggressive diabetic phenotype; indeed they were more likely to require future insulin therapy respect to GDM-miR-330^low^ whose glycemic control was guaranteed by diet indications only; moreover, the rate of those GDM patients having one or more T2D relatives was higher in GDM-miR-330^high^ (Table 3). Although such results were not statistically significant, a secondary indication of a worse diabetic outcome in GDM-miR-330^high^ with respect to GDM-miR-330^low^ was indirectly measured by cesarean section rate [a gestational diabetic complication, strongly related to hyperglycemia and glycemic control during pregnancy (48)], which was significantly higher in GDM-miR-330^high^ thus further confirm a correlation between miR-330-3p plasmatic levels and GDM outcome during pregnancy and reinforcing a potential view of miR-330-3p as an indicator of an aggressive diabetic phenotype. Overall, such results pointed out a potential role for miR-330-3p as a new biomarker of GDM outcome. One potential limit of this study is the small number of patients; however, the use of a specific and stringent procedure for blood drawn, plasma collection, and miRNAs analysis (previously reported as critical for low variance maintenance among samples), provided enough accuracy to obtain statistically significant results, at least for miRNA miR-330-3p. Therefore, although further studies are needed in order to deepen these results, the measurement of circulating levels of miR-330-3p may help in advance to guide the choice of a personalized therapy in GDM, being miR-330-3p circulating levels able to potentially predict diabetic outcome and/or severity.

Although circulating miRNAs could be used as new disease biomarkers, a potential role as mediators of tissue crosstalk has been assigned to their function. Indeed, several authors started to link circulating miRNAs alterations as a putative disruption of a communication track among tissues/cells. Therefore, we hypothesized that the alteration of miR-330-3p expression levels in plasma of GDM patients may be involved in the loss of compensatory potential by beta-cells during pregnancy, thus contributing to GDM onset. miR-330-3p has been previously demonstrated to be active as a cell cycle suppressor during cancer; indeed, the alteration of its expression may cause changes in proliferation and growth thus rendering it a pivotal regulator of cell cycle homeostasis. It is conceivable that miR-330-3p upregulation in plasma of GDM patients, if transferred to beta-cells, may partly contribute to beta-cell dysfunction thus causing defects in proliferation and growth. Indeed, miR-330-3p target genes search using both algorithms for previously experimentally validated targets and those for the prediction revealed that E2F1 and CDC42 are miR-330-3p target genes. Both have been previously demonstrated to be involved in beta-cell proliferation and growth and, moreover, strong evidences indicated them as involved in the control of insulin secretion. Therefore, the reduction of E2F1 and CDC42, potentially caused by increased levels of miR-330-3p, may lead to an impairment of beta-cell proliferation and insulin secretion. In support of this hypothesis, it has been previously demonstrated that cell cycle genes are necessary to beta-cell compensatory adaptation during pregnancy, being 67 those genes involved in proliferation control and upregulated in pregnant mouse islets (49, 50), and that the disruption of their network may cause massive defects in beta-cell adaptation mechanisms. Additionally, beside the control of beta-cell proliferation, Targetscan7.0 miR-330-3p predicted targets, revealed several other genes potentially involved in beta-cell function such as AGTR2 (Angiotensin II receptor Type 2) [which partially CTR beta-cell differentiation (37, 38)] or EIF4EBP2 [involved in beta-cell proliferation and differentiation (40)]. Of particular interest, AGTR2 has been previously demonstrated to be an active factor which exploits its function during pancreas development at embryonic stage, but also addressed as potential mediator of beta-cell regeneration in adult pancreas; it is possible to hypothesize that high plasma levels of miR-330-3p may inhibit pancreatic endocrine/beta-cell neogenesis through the downregulation of AGTR2 expression, thus determining a defect in the regenerative capacity of endocrine pancreas during high metabolic requirements.

Finally, one potential open question remains about determination of circulating miR-330-3p tissue/cells of origin; such information could be of fundamental importance in order to address whether a specific “mirroring” plasma-tissue may be identified. By interrogating several miRNAs expression databases reporting their high-throughput expression in several human tissues, we found that miR-330-3p resulted enriched in breast tissue respect to the other tissues analyzed. Although such data need to be confirmed by other independent observations, a potential indication about its primary derivation sites has been raised, thus opening to the possibility of a miRNA-mediated crosstalk between breast tissue and endocrine pancreas during normal and diabetes-complicated pregnancy.

In conclusion, we demonstrated that miRNA miR-330-3p is upregulated in plasma of GDM patients and identifies a subgroup of those patients with a more adverse course of gestational diabetes. Finally, we suggest that miR-330-3p could be potentially used both as a biomarker of GDM outcome as well as a therapeutic target. However, additional studies are needed to finally confirm the potential use of circulating miRNAs as biomarkers and to uncover their role as dysfunction mediators in GDM.

## Ethics Statement

This study was carried out in accordance with the required local ethical recommendations (University of Siena). All procedures followed were in accordance with the ethical standards of the responsible committee on human experimentation (institutional and national) and with the Helsinki Declaration of 1975, as revised in 2013. Informed consent was obtained from all patients for being included in the study.

## Author Contributions

GS performed the experiments, collected, analyzed, and discussed data, and contributed to write the manuscript. EG recruited patients, collected blood, clinical data, and parameters, and contributed to writing the manuscript. GG performed the experiments and collected and analyzed data. CF recruited patients and collected blood and clinical data parameters. CDP assisted with sample processing and experiments and contributed to scientific discussion. EC collected clinical data parameters and contributed to scientific discussion. FD designed and supervised the study and wrote the manuscript.

## Conflict of Interest Statement

The authors declare that the research was conducted in the absence of any commercial or financial relationships that could be construed as a potential conflict of interest. The reviewer RI and handling Editor declared their shared affiliation.

## Footnotes

^1^http://www.targetscan.org/vert_71/.

^2^http://mirtarbase.mbc.nctu.edu.tw/.

^3^http://thebiogrid.org.
