# Supplementary material for: Circulating microRNA (miRNA) Expression Profiling in Plasma of Patients with Gestational Diabetes Mellitus Reveals Upregulation of miRNA miR-330-3p
Source: Front Endocrinol (Lausanne). 2017 Dec 12;8:345. doi: 10.3389/fendo.2017.00345 (PMC5732927; doi:10.3389/fendo.2017.00345)
Supplement: Supplementary file 1 [file Image_1.PDF]

Supplementary Material

Circulating microRNAs expression profile in plasma of patients with Gestational Diabetes Mellitus

Supplementary Figure 1

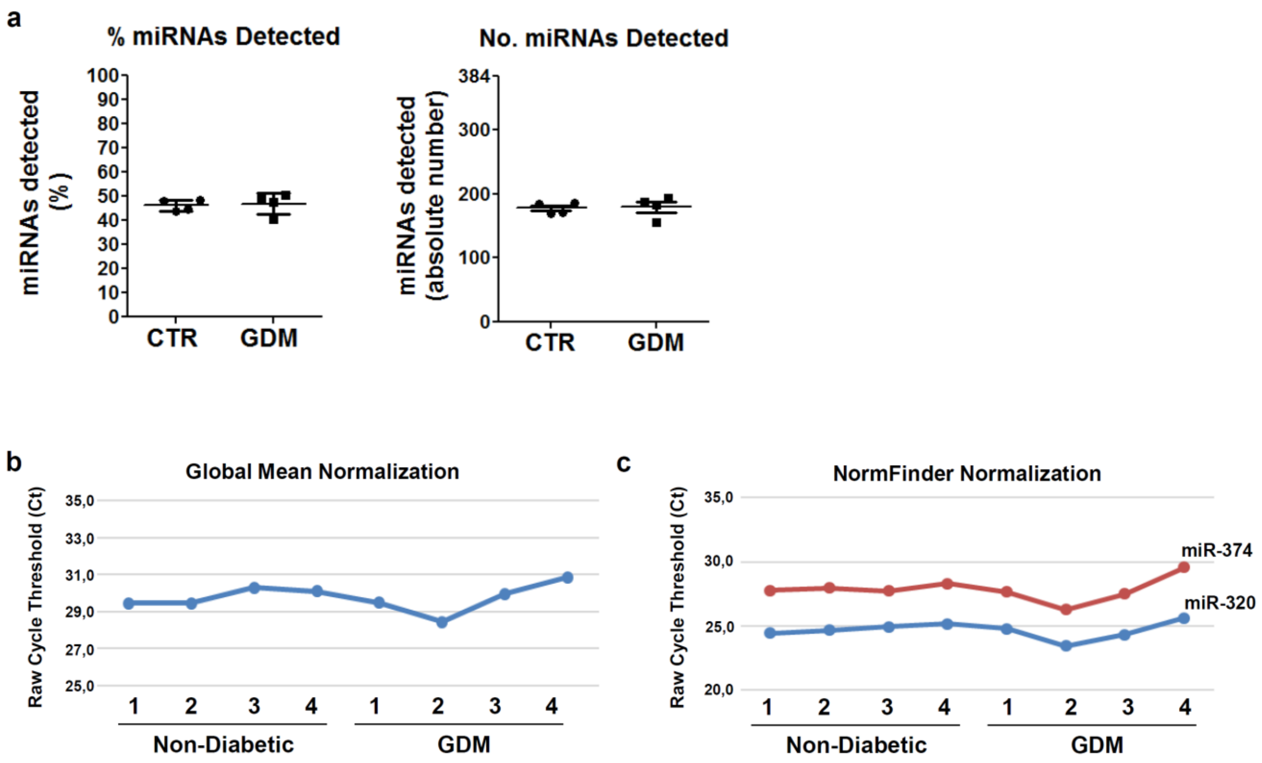

Supplementary Figure 1. MicroRNAs detection rate and normalization strategy. (**A**) Detected microRNAs percentage (left panel) and absolute number (right panel) among 384 microRNAs analyzed in n=4 non-diabetic controls and n=4 GDM patients plasma samples. (**B**) Stability graph of Global Mean Normalization (reported as median Ct) across 8 plasma samples analyzed. (**C**) Stability graph of housekeeping microRNAs miR-320 and miR-374a identified using NormFinder algorithm; data are reported as raw Cycle Threshold (Ct).
